# External validation of the NOBLADS score, a risk scoring system for severe acute lower gastrointestinal bleeding

**DOI:** 10.1371/journal.pone.0196514

**Published:** 2018-04-26

**Authors:** Tomonori Aoki, Atsuo Yamada, Naoyoshi Nagata, Ryota Niikura, Yoshihiro Hirata, Kazuhiko Koike

**Affiliations:** 1 Department of Gastroenterology, Graduate School of Medicine, The University of Tokyo, Tokyo, Japan; 2 Department of Gastroenterology and Hepatology, National Center for Global Health and Medicine, Tokyo, Japan; Duke-NUS Medical School, SINGAPORE

## Abstract

**Background:**

We aimed to evaluate the generalizability of NOBLADS, a severe lower gastrointestinal bleeding (LGIB) prediction model which we had previously derived when working at a different institution, using an external validation cohort. NOBLADS comprises the following factors: non-steroidal anti-inflammatory drug use, no diarrhea, no abdominal tenderness, blood pressure ≤ 100 mmHg, antiplatelet drug use, albumin < 3.0 g/dL, disease score ≥ 2, and syncope.

**Methods:**

We retrospectively analyzed 511 patients emergently hospitalized for acute LGIB at the University of Tokyo Hospital, from January 2009 to August 2016. The areas under the receiver operating characteristic curves (ROCs-AUCs) for severe bleeding (continuous and/or recurrent bleeding) were compared between the original derivation cohort and the external validation cohort.

**Results:**

Severe LGIB occurred in 44% of patients. Several clinical factors were significantly different between the external and derivation cohorts (p < 0.05), including background, laboratory data, NOBLADS scores, and diagnosis. The NOBLADS score predicted the severity of LGIB with an AUC value of 0.74 in the external validation cohort and one of 0.77 in the derivation cohort. In the external validation cohort, the score predicted the risk for blood transfusion need (AUC, 0.71), but was not adequate for predicting intervention need (AUC, 0.54). The in-hospital mortality rate was higher in patients with a score ≥ 5 than in those with a score < 5 (AUC, 0.83).

**Conclusions:**

Although the external validation cohort clinically differed from the derivation cohort in many ways, we confirmed the moderately high generalizability of NOBLADS, a clinical risk score for severe LGIB. Appropriate triage using this score may support early decision-making in various hospitals.

## Introduction

Acute lower gastrointestinal bleeding (LGIB) is a common indication for hospital admission in the United States[1] and accounts for approximately 35.7 per 100,000 adult hospitalizations annually. Although the rate of upper gastrointestinal bleeding (UGIB) has decreased rapidly over the past 10 years[2], the incidence of LGIB has increased slightly[2], and a similar trend has been reported in Asia.[3,4] Patients with acute LGIB often experience persistent or recurrent bleeding and require blood transfusions, long hospitalization stays, and interventions such as colonoscopic, radiological, and surgical treatment.[5–7] In addition, a certain proportion of patients with acute LGIB die during their hospital stay (< 4%).[7,8] Therefore, for appropriate triage to emergency hospitalization or early intervention, and for ultimately better outcomes, a risk stratification tool to predict severe LGIB is required. However, unlike UGIB[9], predictive clinical scores with high generalizability have not been established for severe acute LGIB.

Although some studies have investigated predictors for severe acute LGIB[10–13], few predictive scores have been validated in different settings. Strate et al. validated their score, but only 39% of patients (n = 107) in the validation study were recruited from a hospital other than where the score was developed.[11] Using an external cohort (n = 172), Newman et al. assessed the utility of the BLEED criteria, which suggested predictors for poor outcomes of GI bleeding, but the criteria were not very useful because the area under the receiver operating characteristic curve (ROC-AUC) was relatively low (0.60).[13]

We have recently developed and prospectively validated NOBLADS, a clinical risk scoring system for severe LGIB.[14] However, our validation study was conducted at the same hospital where we developed the score and the number of patients was relatively small (n = 161). Thus, it remains to be determined whether this model can be generalized to other hospitals and to other patients. Because the background, diagnosis, and management of acute LGIB may vary according to different institutions, the ability of this model to provide accurate predictions at other institutions needs to be confirmed.

To evaluate the generalizability of the NOBLADS score for severe LGIB, we investigated an external validation cohort, which was composed of a large number of patients who were hospitalized for acute overt LGIB on an emergency basis.

## Materials and methods

### Study design, setting, and participants

**Fig 1:**
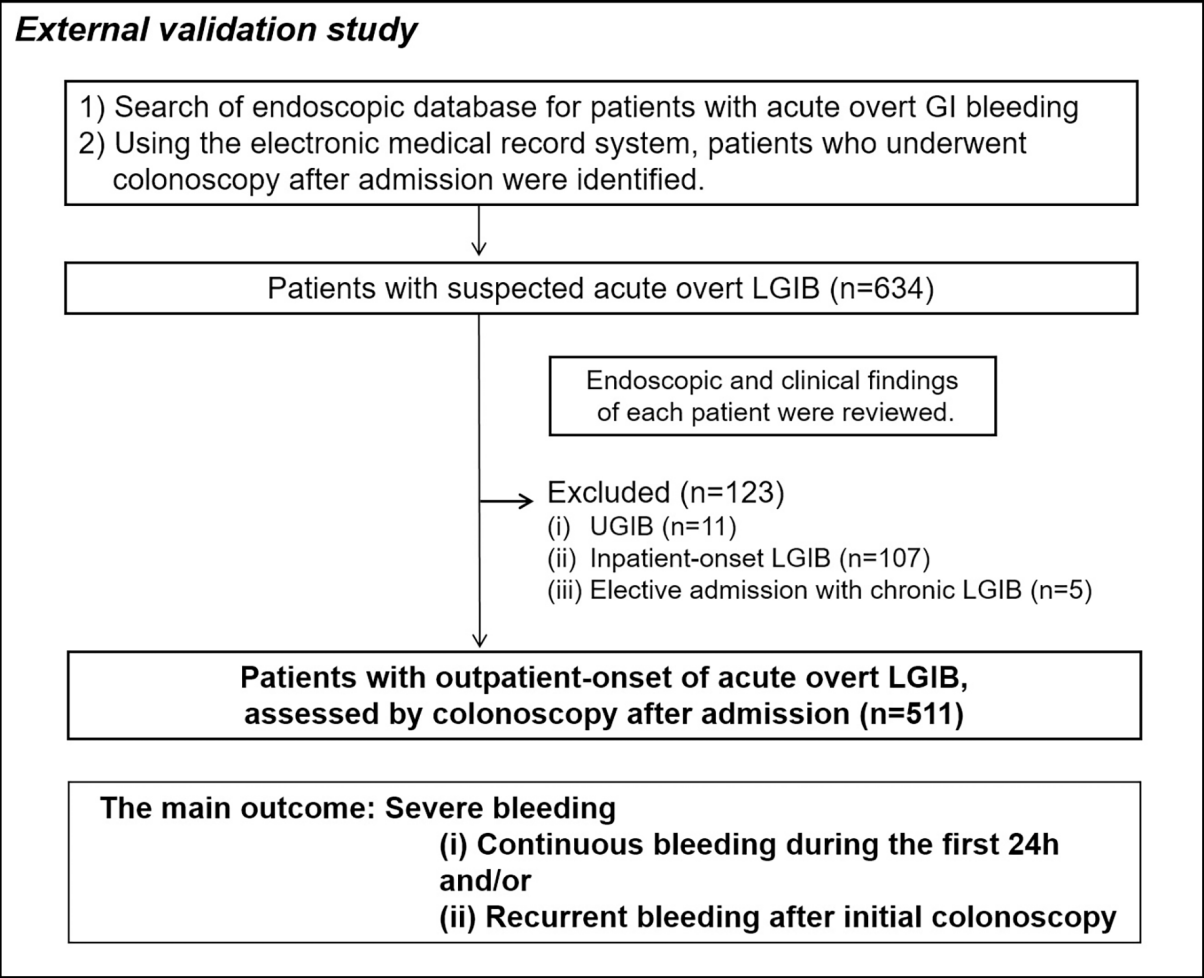
Flow chart of patient selection. LGIB, lower gastrointestinal bleeding; UGIB, upper gastrointestinal bleeding.

This study complied with the Declaration of Helsinki. The design was approved by the ethics committee of The University of Tokyo (approval number 11528) and by the institutional review board at the National Center for Global Health and Medicine (approval number 2163). This study was a retrospective observational study, carried out by the opt-out method of our hospital website. We retrospectively identified patients who were admitted to the University of Tokyo Hospital on an emergency basis due to the onset of acute, continuous, or frequent overt LGIB between January 2009 and August 2016. This hospital is one of the referral university hospitals in the Tokyo metropolitan area, and it is a different institution from the hospital where we developed a clinical risk score for severe LGIB, NOBLADS.[14] Data were collected from admission databases and a recorded endoscopic database. The endoscopic database is a searchable collection of records into which endoscopists prospectively input data after completing endoscopies. We searched the endoscopic database and selected patients with overt GI bleeding who were assessed by colonoscopy (**Fig 1**). We subsequently reviewed the endoscopic and clinical findings of these patients using the electronic medical record system and excluded patients with (i) UGIB, (ii) inpatient-onset LGIB, and (iii) elective admission with chronic LGIB. Ultimately, 511 patients with outpatient-onset acute LGIB were analyzed for the external validation of the NOBLADS score.

### Outcome criteria

Outcomes were defined in the derivation and internal validation studies as follows.[14] The main outcome was severe LGIB comprising: (i) continuous bleeding during the first 24 h (transfusion of ≥ 2 units of packed red blood cells and/or a decrease in hematocrit of ≥ 20%) and/or (ii) recurrent bleeding after initial colonoscopy (rectal bleeding accompanied by a further decrease in hematocrit of ≥ 20% and/or additional blood transfusions) as previously described.[10]

Secondary outcomes included blood transfusion requirement, length of stay (LOS), intervention (endoscopy, interventional radiology, or surgery), and in-hospital mortality. Blood transfusion was indicated when hemoglobin levels fell below 7.0 g/dL (or 8.0 g/dL when vital signs were unstable). After spontaneous cessation of bleeding with conservative treatment or hemostasis, all patients were started on a liquid diet and gradually progressed to a solid diet over a period of three days before being discharged. Endoscopic intervention was the first-line treatment when stigmata of recent hemorrhage (SRH) was detected on colonoscopy. Interventional radiology was performed in patients with extreme bleeds that did not resolve with endoscopic treatment. Patients with persistent bleeds after endoscopic treatment and/or interventional radiology were surgically treated. Data concerning death during hospitalization were collected from the medical records and death certificates of the study hospitals.

### Risk scoring system (NOBLADS score)

We previously used multivariate logistic regression to detect risk factors for severe bleeding in a retrospectively collected cohort of 439 patients emergently hospitalized for acute LGIB at the National Center for Global Health and Medicine in Japan, from January 2009 to December 2013 (i.e., derivation cohort). From these data, we developed NOBLADS, a clinical risk scoring system for severe LGIB.[14] This score comprises the following factors: NSAID use, no diarrhea, no abdominal tenderness, blood pressure ≤ 100 mmHg, antiplatelet drug use (non-aspirin), albumin < 3.0 g/dL, disease score ≥ 2 (according to the Charlson comorbidity index), and syncope (**Table 1**). Each predictor was given a weight of 1 point. In this report, we assessed the external validity of the six-level NOBLADS score using 0, 1, 2, 3, 4, or ≥ 5 predictors.

**Table 1:** Multivariate predictors of severe LGIB (NOBLADS factors) as defined in the previous study (n = 439). [14].

Characteristic	Adjusted OR (95% CI)	Coefficient (95% CI)	P
NSAIDs^§^	2.50 (1.28–4.90)	0.92 (0.24–1.59)	**0.008**
No diarrhea	2.24 (1.13–4.42)	0.81 (0.12–1.49)	**0.020**
No abdominal tenderness	2.97 (1.55–5.67)	1.09 (0.44–1.74)	**0.001**
Blood pressure ≤100 mmHg (systolic)	2.34 (1.26–4.35)	0.85 (0.23–1.47)	**0.007**
Antiplatelet drugs (non-aspirin)^¶^	1.97 (1.06–3.66)	0.68 (0.06–1.30)	**0.032**
Albumin <3.0 g/dL	2.94 (1.57–5.49)	1.08 (0.45–1.70)	**0.001**
Disease score ≥2 (Charlson comorbidity index)	1.70 (1.04–2.78)	0.53 (0.04–1.02)	**0.034**
Syncope^†^	2.49 (1.11–5.56)	0.91 (0.11–1.72)	**0.026**

^§^NSAIDs included non-selective NSAIDs and COX-2 inhibitor.

^¶^Antiplatelet drugs (non-aspirin) included clopidogrel, ticlopidine, dipyridamole, cilostazol, sarpogrelate hydrochloride, ethyl icosapentate, dilazep hydrochloride, limaprost alfadex, and beraprost.

^†^Syncope included transient altered mental status defined as a Glasgow Coma Scale **≤**14 or history of syncope.

Abbreviations: CI, confidence interval; LGIB, lower gastrointestinal bleeding; OR, odds ratio; NSAIDs, non-steroidal anti-inflammatory drugs.

### Diagnosis of LGIB and data collection

All patients in this study were assessed by colonoscopy using high-resolution electronic video endoscopes (type PCF-240I, PCF-Q260AI, or PCF-Q260JI; Olympus Optical, Tokyo, Japan) after bowel preparation with a polyethylene glycol solution. If bowel preparation was inadequate, an Olympus Flushing Pump water-jet (Olympus Optical) was applied to improve visualization. Colonoscopy was repeated for a more detailed assessment if colon preparations were insufficient at the first colonoscopy or if rebleeding occurred. The diagnostic criteria for diverticular bleeding were classified as definitive and presumptive.[15] A definitive diagnosis was based on colonoscopic visualization of a colonic diverticulum with SRH such as active bleeding, adherent clot, or a visible vessel. A presumptive diagnosis was based on fresh blood localized at a colonic diverticulum in the presence of a potential bleeding source on complete colonoscopy, or bright red blood in the rectum confirmed by objective color testing and colonoscopy showing a single potential bleeding source in the colon complemented by negative upper or negative capsule endoscopic findings.[15] Overt LGIB of unknown origin or hemorrhoidal bleeding was defined as a clinically significant decrease in hematocrit of ≥ 10% and/or a decrease in hemoglobin levels of ≥ 2 g/dL from baseline[16]. All required variables (symptoms, vital signs, comorbidities, medications, and laboratory findings) were collected in the emergency department within two hours of a patient presenting at our hospital. We evaluated 19 comorbidities using the Charlson comorbidity index.[17]

### Statistics

Characteristics of the derivation cohort and the external validation cohort were compared using a univariate analysis with the Pearson’s Chi-squared test, Fisher’s exact test, or Wilcoxon rank sum test as appropriate. We used previously published data[14] in the derivation cohort (n = 439) and in the internal validation cohort (n = 161) to compare the validity and prediction ability of the NOBLADS score. We assessed the validity of the NOBLADS score using ROC-AUC of the external validation cohort compared with the derivation cohort and the internal validation cohort. Model calibration in the external validation cohort was evaluated using the Hosmer–Lemeshow goodness-of-fit test. The ability of the score to predict severe bleeding and secondary outcomes including blood transfusion requirement, LOS, intervention requirements and in-hospital mortality was determined in the external validation cohort. These relationships were assessed using a nonparametric trend test (*nptrend* in Stata) or Fisher’s exact test.

A value of P < 0.05 was considered to indicate statistical significance. The STATA version 13 software was used to perform all analyses (StataCorp, College Station, TX, USA).

## Results

### Patient characteristics

We analyzed data from 511 patients (male, 66.1%; mean age, 68.7 years; range, 16–99 years) with LGIB (**Table 2**). The external validation cohort and the derivation cohort were different in that the former had a significantly higher number of males, more comorbid diseases, lower initial hematocrit levels, a greater number of blood transfusions, higher NOBLADS scores, and were more often diagnosed with diverticular bleeding than the latter cohort. The two cohorts had similar initial vital signs. According to the NOBLADS factors, rates of no diarrhea, no abdominal tenderness, non-aspirin antiplatelet drug use, and Charlson comorbidity index ≥ 2 were significantly higher in the external validation cohort than in the derivation cohort, whereas rates of NSAID use, blood pressure ≤ 100 mmHg, albumin < 3.0 g/dL, and syncope were similar in the two cohorts.

**Table 2:** Patient characteristics.

Characteristics	Derivation cohort (n = 439)[14]	External validation cohort (n = 511)	P-value
Age ≥ 65 years, n (%)	273 (62.2)	347 (67.9)	0.065
Sex, M, n (%)	241 (54.9)	338 (66.1)	**< 0.001**
Mean disease score (Charlson comorbidity index) (± SD)	1.7 ± 2.0	2.3 ± 2.2	**< 0.001**
Mean heart rate, min (± SD)	84.0 ± 16.0	85.3 ± 17.4	0.544
Mean systolic blood pressure, mmHg (± SD)	126.7 ± 24.3	124.6 ± 23.4	0.164
Requirement of blood transfusion, n (%)	115 (26.2)	212 (41.5)	**< 0.001**
Mean units of blood transfusion, units (± SD)	2.1 ± 4.6	3.5 ± 10.1	**< 0.001**
Mean initial hematocrit, % (± SD)	33.7 ± 8.2	32.8 ± 7.8	**0.027**
NOBLADS factors, n (%)			
NSAIDs^§^	56 (12.8)	53 (10.4)	0.250
No diarrhea	289 (65.8)	471 (92.2)	**< 0.001**
No abdominal tenderness	318 (72.4)	449 (87.9)	**< 0.001**
Blood pressure ≤ 100 mmHg (systolic)	63 (14.4)	78 (15.3)	0.693
Antiplatelet drugs (non-aspirin)^¶^	64 (14.6)	113 (22.1)	**0.003**
Albumin < 3.0 g/dL	68 (15.5)	61 (11.9)	0.111
Syncope^†^	35 (8.0)	35 (6.9)	0.509
Charlson comorbidity index ≥ 2	183 (41.7)	286 (56.0)	**< 0.001**
Diabetes mellitus	71 (16.2)	123 (24.1)	**0.003**
Cerebrovascular disease	66 (15.0)	74 (14.5)	0.811
Chronic pulmonary disease	10 (2.3)	16 (3.1)	0.422
Connective tissue disease	17 (3.9)	26 (5.1)	0.369
Myocardial infarction	69 (15.7)	132 (25.8)	**< 0.001**
Ulcer disease	44 (10.0)	54 (10.6)	0.783
Chronic kidney disease	99 (22.6)	114 (22.3)	0.929
Liver cirrhosis	24 (5.5)	30 (5.9)	0.789
Malignancy	72 (16.4)	115 (22.5)	**0.018**
NOBLADS score, n (%)			**< 0.001**
0	50 (11.4)	11 (2.2)	
1	60 (13.7)	31 (6.1)	
2	115 (26.2)	136 (26.6)	
3	115 (26.2)	145 (28.4)	
4	62 (14.1)	142 (27.8)	
≥ 5	37 (8.4)	46 (9.0)	
Mean scores (± SD)	2.5 ± 1.5	3.0 ± 1.2	
Diagnosis, n (%)			
Diverticular bleeding	202 (46.0)	310 (60.7)	**< 0.001**
Definitive diagnosis	45 (10.3)	76 (14.9)	**0.033**
Presumptive diagnosis	157 (35.8)	234 (45.8)	**0.002**
Ischemic colitis	84 (19.1)	24 (4.7)	**< 0.001**
Inflammatory bowel disease	24 (5.5)	12 (2.4)	**0.012**
Infectious colitis	17 (3.9)	10 (2.0)	0.077
Colorectal cancer	17 (3.9)	6 (1.2)	**0.007**
Post-polypectomy bleeding	17 (3.9)	46 (9.0)	**0.002**
Hemorrhoid	14 (3.2)	5 (1.0)	**0.015**
Angioectasia/angiodysplasia	14 (3.2)	10 (2.0)	0.228
Radiation proctitis	8 (1.8)	6 (1.2)	0.408
Small intestinal hemorrhage	7 (1.6)	20 (3.9)	**0.032**
Rectal ulcer	6 (1.4)	8 (1.6)	0.800
Drug-induced ulcer	5 (1.1)	4 (0.8)	0.572
Non-specific colitis	4 (0.9)	8 (1.6)	0.368
Unknown ulcer	2 (0.5)	5 (1.0)	0.347
Miscellaneous*	8 (1.8)	12 (2.4)	0.451
Unknown**	10 (2.3)	24 (4.7)	**0.045**

^§^NSAIDs included non-selective NSAIDs and COX-2 inhibitors.

^¶^Antiplatelet drugs (non-aspirin) included clopidogrel, ticlopidine, dipyridamole, cilostazol, sarpogrelate hydrochloride, ethyl icosapentate, dilazep hydrochloride, limaprost alfadex, and beraprost.

^†^Syncope included transient altered mental status defined by a Glasgow Coma Scale score ≤ 14 or history of syncope.

*Miscellaneous cases included bleeding from polyps, malignancy other than colorectal cancer, bleeding after prostate biopsy, and anastomotic varices.

**Negative for UGIB, confirmed with upper gastrointestinal endoscopy.

Abbreviations: M, male; SD, standard deviation; NSAIDs, non-steroidal anti-inflammatory

Bleeding was severe in 44% of the external cohort. Severe LGIB included only continued bleeding (n = 193), only recurrent bleeding (n = 17), and both (n = 15). A total of 406 patients (79%) were assessed using early colonoscopy within 24 hours of admission and 105 patients (21%) were assessed using elective colonoscopy. The mean length of stay was 11.9 ± 11.2 days. One hundred and forty-four patients (28%) underwent interventions as follows: 135 (26%), endoscopy; one (0.2%), radiology; and eight (1.6%), surgery.

### Evaluation of the NOBLADS score in the external validation cohort

**Fig 2:**
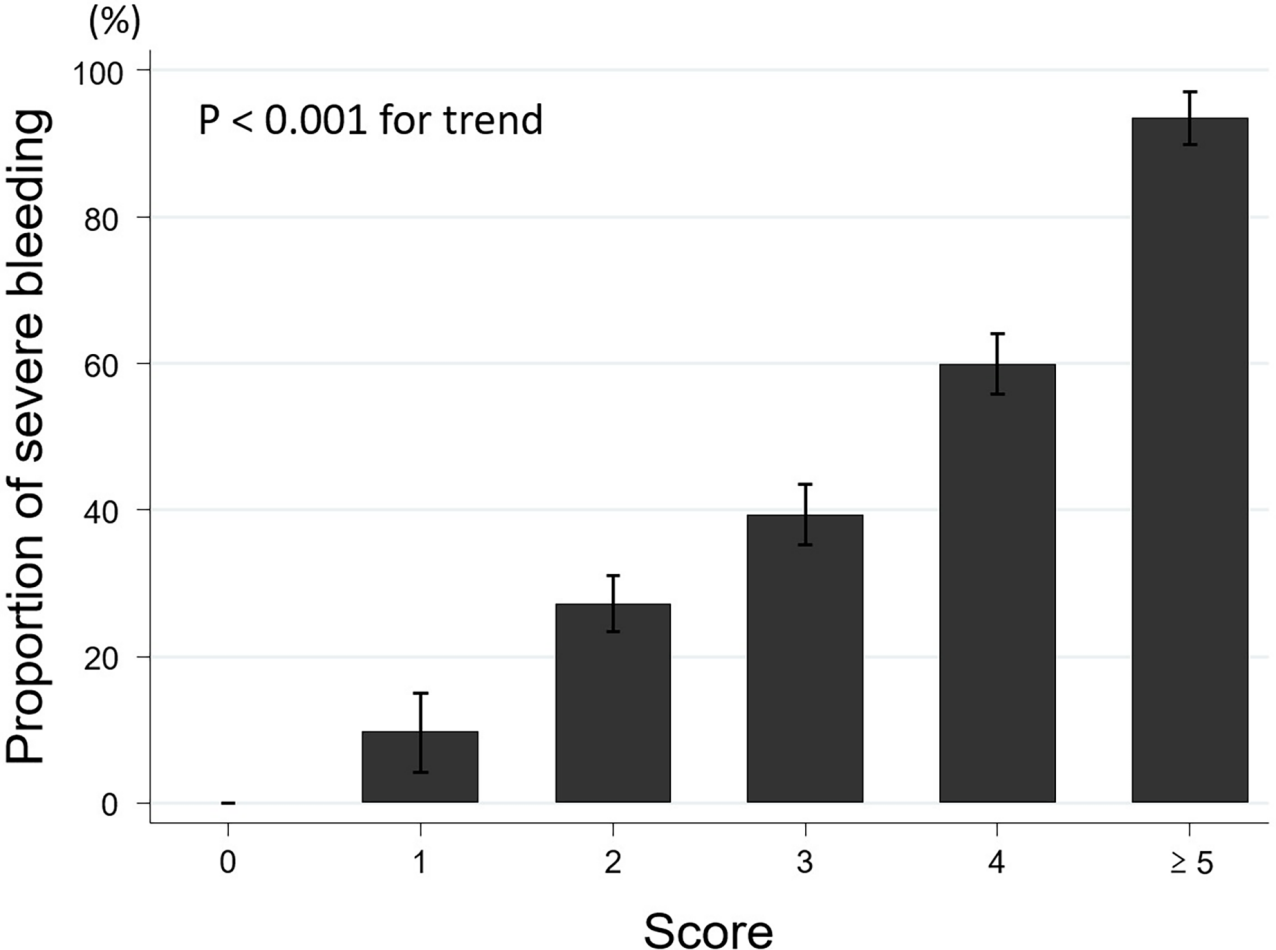
Prediction of severe bleeding using the NOBLADS score in the external validation cohort. Error bars show the standard errors.

**Fig 3:**
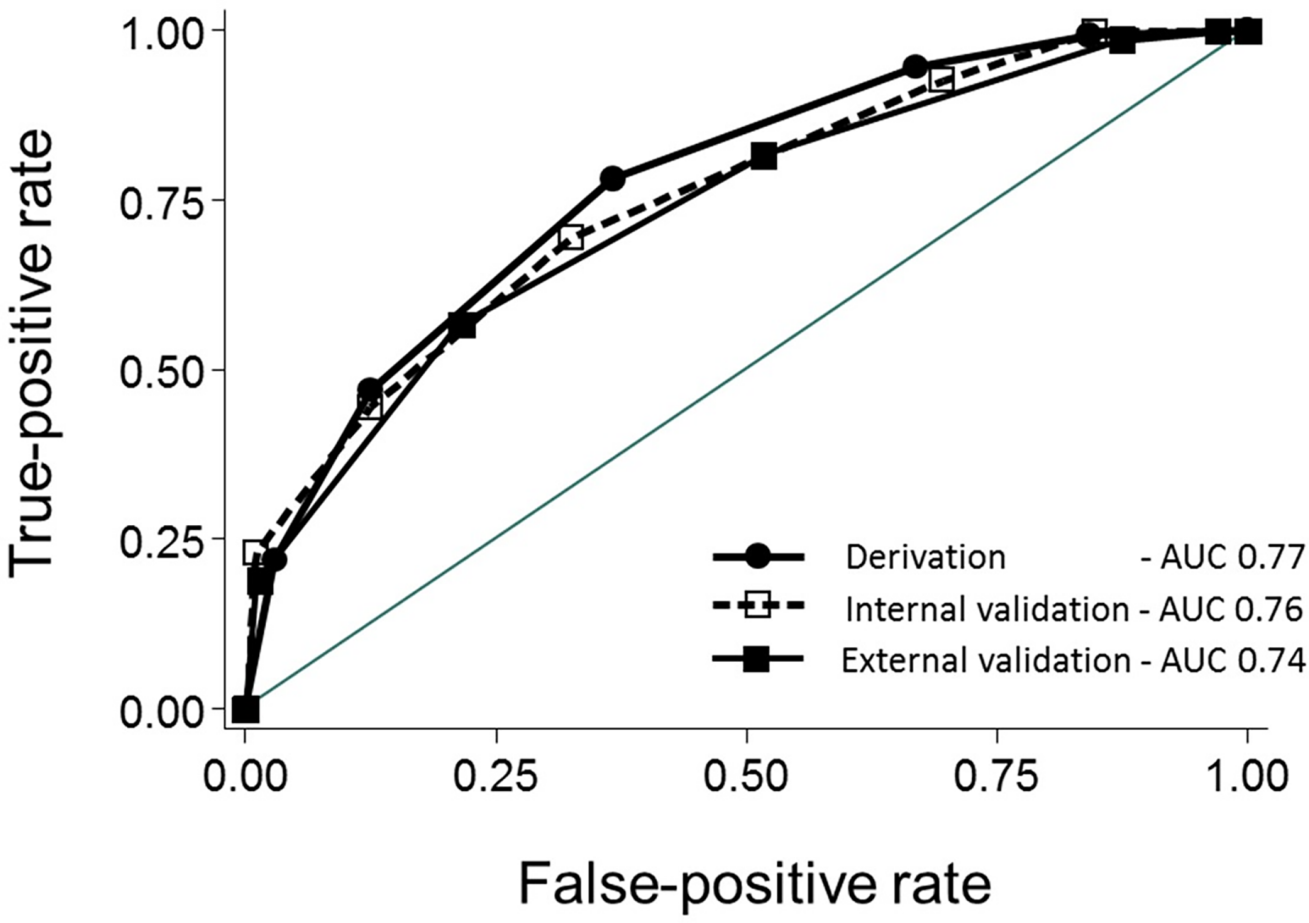
Receiver operator characteristic curves of NOBLADS scores for severe LGIB in the derivation (n = 439)[14], internal validation (n = 161)[14], and external validation cohorts (n = 511). AUC, area under receiver operating characteristic curve.

The rates of severe bleeding with 0, 1, 2, 3, 4, and ≥ 5 predictors were 0% (0/11), 9.7% (3/31), 27.2% (37/136), 39.3% (57/145), 59.9% (85/142), and 93.5% (43/46), respectively (P < 0.001, trend) (**Fig 2**) and the AUC of NOBLADS for the external validation set was 0.74 (95% CI, 0.70–0.78; **Fig 3**). The P value was 0.087 (> 0.05) for the Hosmer–Lemeshow test.

**Fig 4:**
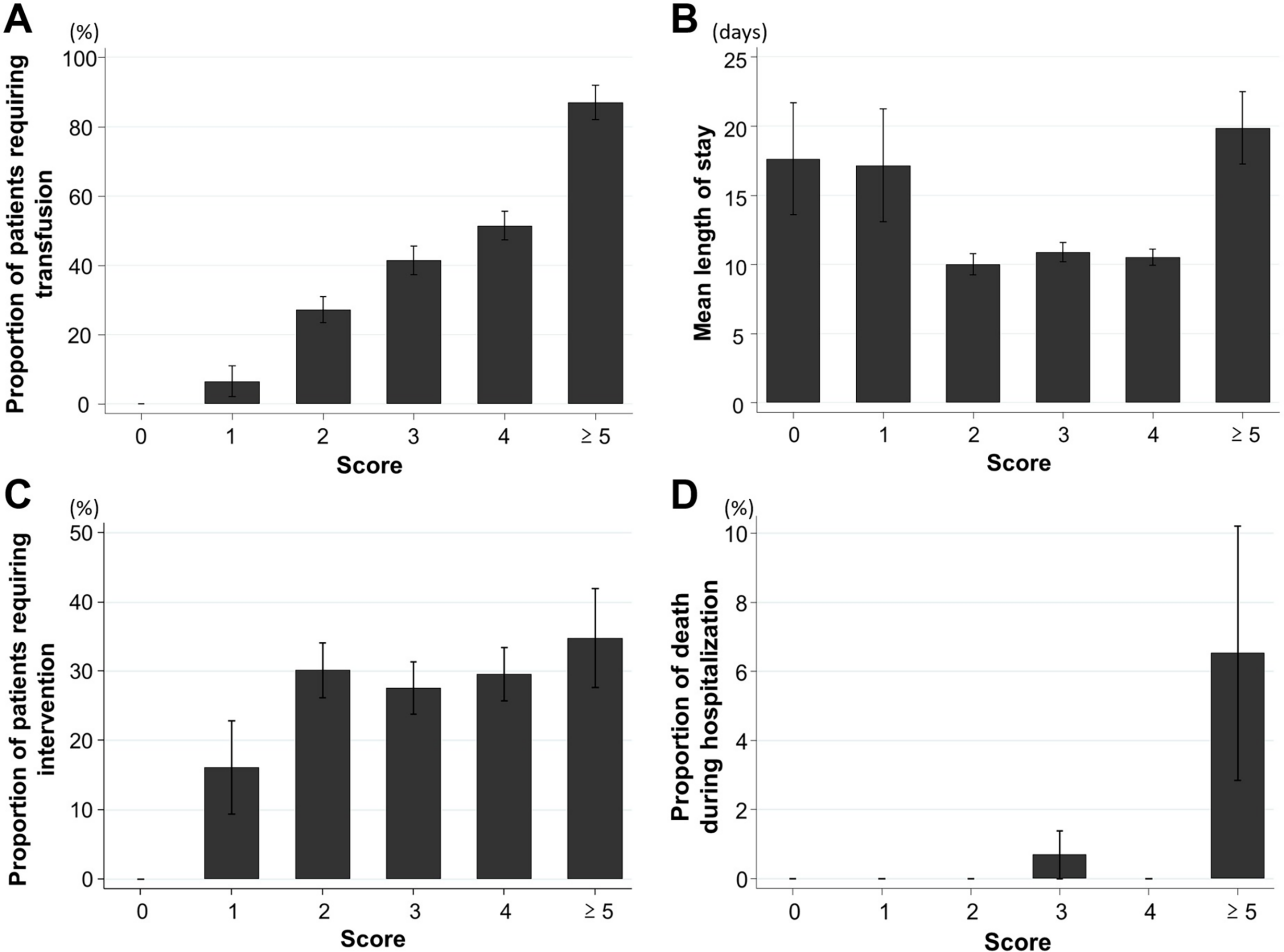
Prediction of adverse clinical outcomes using the NOBLADS score in the external validation cohorts. A, proportion of patients who required blood transfusions; B, mean length of stay in hospital; C, proportion of patients who required intervention; and D, proportion of death during hospitalization. Error bars show the standard errors.

Among the patients with 0, 1, 2, 3, 4, and ≥ 5 predictors, the rates of blood transfusion requirements were 0%, 6.5%, 27.2%, 41.4%, 51.4% and 87.0% (P < 0.001, trend; AUC, 0.71) (**Fig 4A**); the mean units of blood transfusion were 0, 0.3, 2.0, 3.6, 3.3, and 11.0 (P < 0.001, trend); the mean LOS were 17.6, 17.2, 10.0, 10.9, 10.5 and 19.9 days (P = 0.014, trend) (**Fig 4B**); and rates of required intervention were 0%, 16.1%, 30.2%, 27.6%, 29.6% and 34.8%, respectively (P = 0.060, trend; AUC, 0.54) (**Fig 4C**).

Four patients (0.8%) died during hospitalization. Of these, two deaths were related to GI bleeding, whereas the other two were not related to GI bleeding (multiple myeloma and malignant lymphoma). The rates of death with 0, 1, 2, 3, 4, and ≥ 5 predictors were 0% (0/11), 0% (0/31), 0% (0/136), 0.7% (1/145), 0% (0/142), and 6.5% (3/46), respectively (**Fig 4D**). The mortality rate of patients with a score ≥ 5 (6.5%) was significantly higher than that of patients with a score < 5 (0.2%) (P < 0.001, trend; AUC, 0.83).

## Discussion

We validated the usefulness of the NOBLADS score, which consists of eight predictors, in a relatively large group of acute LGIB patients. Although many clinical factors including background, laboratory data, NOBLADS factors, and endoscopic diagnosis differed between the external and derivation cohorts, the predictive accuracy of the score was moderately high in the external validation cohort (AUC, 0.74) as well as in the derivation cohort (AUC, 0.77).[14] The score predicted the risk for blood transfusion requirement and in-hospital mortality, but was not able to predict longer hospital stay and intervention, in this external validation cohort.

As shown in Table 1, the external validation cohort appeared to have more patients with a high vascular event risk and with more severe bleeding, compared with the derivation cohort. Moreover, the distribution of diseases differed somewhat between these two cohorts. As shown in Figs 2 and 3, the NOBLADS score effectively predicted severe bleeding in a cohort who were more unwell, and with more comorbidities, than an earlier cohort. This indicates the high generalizability of the score.

Although the p value for trend was significant in the analysis of LOS, caution should be exercised for the interpretation of this result because the mean LOS of patients with low-risk scores (0–1) was longer than that of patients with higher scores (2–4) (**Fig 4B**). One reason might be that low-risk scores (0–1) included 67% of patients with inflammatory bowel disease. The disease tended to require longer hospital stays for remission induction treatments such as corticosteroids. In addition, in this cohort, the NOBLADS score could not significantly predict intervention (p = 0.060, for trend; AUC, 0.54). Thus, further data are needed to determine if this score can be widely used to predict these important outcomes, although the score predicted these outcomes in the original study[14]. Particularly for intervention, an appropriate predictive model is needed, because all seven previous models failed to predict intervention in a recent LGIB study.[18]

The mortality rate of patients with a score ≥ 5 was 6.5% (3/46), whereas that of patients with a score < 5 was 0.2% (1/465). Although the in-hospital mortality of LGIB is generally low[7,8], our results suggest that patients with NOBLADS scores ≥ 5 should be monitored closely, with appropriate allocation of resources, given their high risk of in-hospital mortality. As NOBLADS includes the Charlson comorbidity index[17,19], hypoalbuminemia[19,20], and NSAID use[7,8], which were earlier established as mortality predictors, the NOBLADS score may predict in-hospital mortality. Recently, Sengupta et al.[19] derived and validated a prognostic score predicting LGIB mortality. In our external validation cohort, the AUC of the score by Sengupta et al. was 0.95. Because there were only a few events in this study, further data is needed to validate the utility of using NOBLADS to predict mortality.

Previously, some studies investigated predictors of severe acute LGIB[10–13, 18, 21–23], and a few scores have been validated in other settings. The BLEED criteria[21] failed to be validated in other LGIB patients (AUC, 0.60).[13] The score by Strate et al.[11] may not be generalizable, because more than half of the patients in the validation study were recruited from the same hospital where the score was developed. Although Das et al. validated an artificial neural network (ANN) model[22], it is cumbersome and requires the entry of as many as 26 variables. The scores by Velayos et al.[12], Newman et al.[13], and Chong et al.[23] lack validation. Recently, the score by Oakland et al.[18] afforded a better discriminative performance to identify patients for safe outpatient management than the NOBLADS score and other predictive models. The main difference between the NOBLADS score and the score by Oakland et al.[18] is that the hemoglobin level was the most important predictor in the score by Oakland et al. [18]. Prospective comparisons are needed to determine which scoring system (with or without the hemoglobin level) performs best.

Our study had some limitations. First, this study was retrospectively designed. Second, even though the patient populations were distinct, this external validation study was not fully independent from the derivation study since the same investigators performed both studies. Third, because both the derivation and validation cohorts of this score did not include inpatient-onset patients and patients who were discharged from the emergency room, it is unclear if the NOBLADS score can be applied to these patients. Fourth, the inclusion criteria for patients who underwent colonoscopy might have caused selection bias. In Japan, most institutions including our hospital perform endoscopies at first for diagnosis and treatment for almost all of the GIB patients. We did not perform colonoscopy in few cases such as patients who had already undergone colonoscopy for GIB in the past few months, or in patients whose activities of daily living were too low for colonoscopy. Therefore, it is also unclear whether this score can be applied to these patients. Our study also had strengths. For example, by only including patients who underwent colonoscopy, we were able to measure the rate of intervention. Furthermore, we evaluated the usefulness of the NOBLADS score using patients whose characteristics varied widely from the derivation cohort, and that the sample size was larger (n = 511) than that of previous validation studies[11,22] (n ≤ 252).

In conclusion, we externally validated the NOBLADS, a clinical risk score for severe LGIB. This score may guide a standardized approach in managing acute LGIB. Further prospective studies in other countries are warranted to examine whether the consistent application of this score to LGIB management can reduce adverse outcomes and resource utilization.

## Abbreviations

ROC-AUC: area under the receiver operating characteristic curve
CI: confidence interval
LGIB: lower gastrointestinal bleeding
LOS: length of stay
NCGM: National Center for Global Health and Medicine
NSAIDs: nonsteroidal anti-inflammatory drugs
OR: odds ratio
IQR: interquartile range
SD: standard deviation
UGIB: upper gastrointestinal bleeding

## References

[1] Longstreth GF. Colonoscopy and lower GI bleeding. Am J Gastroenterol. 2002;97: 203–204. doi: 10.1111/j.1572-0241.2002.05402.x 11808952 10.1111/j.1572-0241.2002.05402.x

[2] Lanas A, Garcia-Rodriguez LA, Polo-Tomas M, Ponce M, Alonso-Abreu I, Perez-Aisa MA, et al Time trends and impact of upper and lower gastrointestinal bleeding and perforation in clinical practice. Am J Gastroenterol. 2009;104: 1633–1641. doi: 10.1038/ajg.2009.164 19574968 10.1038/ajg.2009.164

[3] Miyamoto M, Haruma K, Okamoto T, Higashi Y, Hidaka T, Manabe N. Continuous proton pump inhibitor treatment decreases upper gastrointestinal bleeding and related death in rural area in Japan. J Gastroenterol Hepatol. 2012;27: 372–377. doi: 10.1111/j.1440-1746.2011.06878.x 21793917 10.1111/j.1440-1746.2011.06878.x

[4] Nagata N, Niikura R, Aoki T, Shimbo T, Itoh T, Goda Y, et al Increase in colonic diverticulosis and diverticular hemorrhage in an aging society: lessons from a 9-year colonoscopic study of 28,192 patients in Japan. Int J Colorectal Dis. 2014;29: 379–385. doi: 10.1007/s00384-013-1808-4 24317937 10.1007/s00384-013-1808-4

[5] Nagata N, Niikura R, Sakurai T, Shimbo T, Aoki T, Moriyasu S, et al Safety and Effectiveness of Early Colonoscopy in Management of Acute Lower Gastrointestinal Bleeding on the Basis of Propensity Score Matching Analysis. Clin Gastroenterol Hepatol. 2016;14: 558–564. doi: 10.1016/j.cgh.2015.10.011 26492844 10.1016/j.cgh.2015.10.011

[6] Strate LL, Gralnek IM. ACG Clinical Guideline: Management of Patients With Acute Lower Gastrointestinal Bleeding. Am J Gastroenterol. 2016. 10.1038/ajg.2016.41 PMC5099081 26925883

[7] Niikura R, Yasunaga H, Yamaji Y, Horiguchi H, Fushimi K, Yamada A, et al Factors affecting in-hospital mortality in patients with lower gastrointestinal tract bleeding: a retrospective study using a national database in Japan. J Gastroenterol. 2015;50: 533–540. doi: 10.1007/s00535-014-0994-3 25181990 10.1007/s00535-014-0994-3

[8] Strate LL, Ayanian JZ, Kotler G, Syngal S. Risk factors for mortality in lower intestinal bleeding. Clin Gastroenterol Hepatol. 2008;6: 1004–10; quiz 955-. doi: 10.1016/j.cgh.2008.03.021 18558513 10.1016/j.cgh.2008.03.021 PMC2643270

[9] Blatchford O, Murray WR, Blatchford M. A risk score to predict need for treatment for upper-gastrointestinal haemorrhage. Lancet. 2000;356: 1318–1321. doi: 10.1016/S0140-6736(00)02816-6 11073021 10.1016/S0140-6736(00)02816-6

[10] Strate LL, Orav EJ, Syngal S. Early predictors of severity in acute lower intestinal tract bleeding. Arch Intern Med. 2003;163: 838–843. doi: 10.1001/archinte.163.7.838 12695275 10.1001/archinte.163.7.838

[11] Strate LL, Saltzman JR, Ookubo R, Mutinga ML, Syngal S. Validation of a clinical prediction rule for severe acute lower intestinal bleeding. Am J Gastroenterol. 2005;100: 1821–1827. doi: 10.1111/j.1572-0241.2005.41755.x 16086720 10.1111/j.1572-0241.2005.41755.x

[12] Velayos FS, Williamson A, Sousa KH, Lung E, Bostrom A, Weber EJ, et al Early predictors of severe lower gastrointestinal bleeding and adverse outcomes: a prospective study. Clin Gastroenterol Hepatol. 2004;2: 485–490. 15181617 10.1016/s1542-3565(04)00167-3

[13] Newman J, Fitzgerald JE, Gupta S, von Roon AC, Sigurdsson HH, Allen-Mersh TG. Outcome predictors in acute surgical admissions for lower gastrointestinal bleeding. Colorectal Dis. 2012;14: 1020–1026. doi: 10.1111/j.1463-1318.2011.02824.x 21910819 10.1111/j.1463-1318.2011.02824.x

[14] Aoki T, Nagata N, Shimbo T, Niikura R, Sakurai T, Moriyasu S, et al Development and Validation of a Risk Scoring System for Severe Acute Lower Gastrointestinal Bleeding. Clin Gastroenterol Hepatol. 2016. 10.1016/j.cgh.2016.05.042 27311620

[15] Jensen DM, Machicado GA, Jutabha R, Kovacs TO. Urgent colonoscopy for the diagnosis and treatment of severe diverticular hemorrhage. N Engl J Med. 2000;342: 78–82. doi: 10.1056/NEJM200001133420202 10631275 10.1056/NEJM200001133420202

[16] Chan FK, Cryer B, Goldstein JL, Lanas A, Peura DA, Scheiman JM, et al A novel composite endpoint to evaluate the gastrointestinal (GI) effects of nonsteroidal antiinflammatory drugs through the entire GI tract. J Rheumatol. 2010;37: 167–174. doi: 10.3899/jrheum.090168 19884267 10.3899/jrheum.090168

[17] Charlson M, Szatrowski TP, Peterson J, Gold J. Validation of a combined comorbidity index. J Clin Epidemiol. 1994;47: 1245–1251. 7722560 10.1016/0895-4356(94)90129-5

[18] Oakland K, Jairath V, Uberoi R, Guy R, Ayaru L, Mortensen N, et al Derivation and validation of a novel risk score for safe discharge after acute lower gastrointestinal bleeding: a modelling study. Lancet Gastroenterol Hepatol. 2017;2: 635–643. doi: 10.1016/S2468-1253(17)30150-4 28651935 10.1016/S2468-1253(17)30150-4

[19] Sengupta N, Tapper EB. Derivation and Internal Validation of a Clinical Prediction Tool for 30-Day Mortality in Lower Gastrointestinal Bleeding. Am J Med. 2017;130: 601.e1–601.e8. 10.1016/j.amjmed.2016.12.009 28065767

[20] Goldwasser P, Feldman J. Association of serum albumin and mortality risk. J Clin Epidemiol. 1997;50: 693–703. 9250267 10.1016/s0895-4356(97)00015-2

[21] Kollef MH, Canfield DA, Zuckerman GR. Triage considerations for patients with acute gastrointestinal hemorrhage admitted to a medical intensive care unit. Crit Care Med. 1995;23: 1048–1054. 7774215 10.1097/00003246-199506000-00009

[22] Das A, Ben-Menachem T, Cooper GS, Chak A, Sivak MV Jr, Gonet JA, et al Prediction of outcome in acute lower-gastrointestinal haemorrhage based on an artificial neural network: internal and external validation of a predictive model. Lancet. 2003;362: 1261–1266. doi: 10.1016/S0140-6736(03)14568-0 14575969 10.1016/S0140-6736(03)14568-0

[23] Chong V, Hill AG, MacCormick AD. Accurate triage of lower gastrointestinal bleed (LGIB)—A cohort study. Int J Surg. 2016;25: 19–23. doi: 10.1016/j.ijsu.2015.11.003 26612527 10.1016/j.ijsu.2015.11.003

